# Supplementary material for: A Meta-Analysis of the Impact of Nutritional Supplementation on Osteoarthritis Symptoms
Source: Nutrients. 2022 Apr 12;14(8):1607. doi: 10.3390/nu14081607 (PMC9025331; doi:10.3390/nu14081607)
Supplement: Supplementary file 1 [file nutrients-14-01607-s001.zip › nutrients-1641272-supplementary.pdf]

A meta-analysis of the impact of nutritional supplementation on osteoarthritis symptoms.

Supplementary File S1. Equation search terms

1) In Medline

"Vitamins"[Mesh] OR vitamins OR vitamin OR « vitamin B » OR « vitamin C » OR "Vitamin D3 24-Hydroxylase"[Mesh] OR "Sodium-Coupled Vitamin C Transporters"[Mesh] OR "Vitamin D Response Element"[Mesh] OR "Vitamin B 6"[Mesh] OR "Vitamin E"[Mesh] OR "Vitamin D-Binding Protein"[Mesh] OR "Vitamin D"[Mesh] OR "Vitamin B 12"[Mesh] OR "Vitamin A"[Mesh] OR "Calcitriol"[Mesh] OR "Ascorbic Acid Deficiency"[Mesh] OR "aescorin" [Supplementary Concept] OR "Ergosterol"[Mesh] OR "Folic Acid"[Mesh] OR "vitamin D2 glucosiduronate" [Supplementary Concept] OR "vitamin D2 sulfate" [Supplementary Concept] OR "vitamin D3 glucosiduronate" [Supplementary Concept] OR "Tretinoin"[Mesh] OR "Cholecalciferol"[Mesh] OR "1,25-dihydroxy-26-(hydroxymethyl)vitamin D3" [Supplementary Concept] OR "alpha-lipoic acid, 4-aminobenzoic acid, aniline, benfotiamine, thioctic Acid, vitamin E drug combination" [Supplementary Concept] OR "1,25-dihydroxy-5,6-16-ene-vitamin D3" [Supplementary Concept] OR "1,25-dihydroxy-22-ene-vitamin D3" [Supplementary Concept] OR "1,25-dihydroxy-21-(3-hydroxy-3-methylbutyl)vitamin D(3)" [Supplementary Concept] OR "vitamin D 1-alpha hydroxylase" [Supplementary Concept] OR "dihydroxy-vitamin D3" [Supplementary Concept] OR "1,25-dihydroxy-16,23-diene vitamin D3" [Supplementary Concept] OR "Vitamin A"[Mesh] OR "Vitamin A" OR "Tretinoin"[Mesh] OR "Tretinoin" OR "Alitretinoin "[Mesh] OR « Alitretinoin » OR "Vitamin B Complex"[Mesh] OR « vitamin B » OR « vitamin B6 » OR « vitamin B12 » OR « Ascorbic Acid"[Mesh] OR « Ascorbic Acid" OR "Dehydroascorbic Acid" [Mesh] OR "Dehydroascorbic Acid" OR "Vitamin D"[Mesh] OR « Vitamin D » OR "Ergocalciferols"[Mesh] OR « Ergocalciferols » OR "Cholecalciferol"[Mesh] OR « Cholecalciferol » OR "Hydroxycholecalciferols"[Mesh] OR "Dihydroxycholecalciferols"[Mesh] OR "Calcitriol"[Mesh] OR "Vitamin E"[Mesh] OR « Vitamin E » OR "Tocopherols"[Mesh] OR « Tocopherols » OR "Tocotrienols"[Mesh] OR « tocotrienols » OR ("Spices" [Mesh] OR "Spice" OR "Spices" OR "Garlic" [Mesh] OR "Allium" [Mesh] OR "Allium sativum" OR "garlic" OR "Curcuma" [Mesh] AND "Curcumas" OR "Turmeric" OR "Turmerics" OR "Curcuma zedoaria" OR "Curcuma zedoaries" OR "zedoaria, Curcuma" OR "Zedoary zedoaria" OR "Zedoary zedoaries" OR "zedoaria, Zedoary" OR "Curcuma longa" OR "Curcuma longas" OR "longa, Curcuma" OR "Ginger" [Mesh] OR "Gingers" OR "Zingiber officinale" OR "Zingiber officinales" OR "officinales Zingiber" OR "Cinnamomum zeylanicum" [Mesh] OR "Cinnamomum verum" OR "Cinnamon" OR "Cinnamons" OR "Cinnamomum" [Mesh] OR "Cinnamomums" OR "Crocus" [Mesh] OR "Saffron" OR "Saffrons" OR "Crocus sativus" OR "Saffron Crocus" OR "Crocus, Saffron" OR "Iridaceae" [Mesh] OR "Sugar"[Mesh] OR "Sugar" OR "glucose" OR "spice" OR "salt" OR "saline" OR "Fatty Acids, Omega-3" OR "omega 3" OR "n-3 Fatty Acids" OR "n-3 Polyunsaturated Fatty Acid" OR

“n-3 PUFA” OR “Omega 3 Fatty Acids” OR "Fatty Acids, Omega-6" OR “omega 6” OR “n-6 Fatty Acids” OR “n-6 Polyunsaturated Fatty Acid” OR “n-6 PUFA” OR “Omega 6 Fatty Acids” OR “Polyunsaturated Fatty Acids”) AND (“osteoarthritis”[Mesh] OR osteoarthrosis OR osteoarthroses OR “degenerative arthritis”).
Limits: Human and adult

2) In the Cochrane Library

#1 osteoarthritis OR osteoarthrosis
#2 vitamins OR vitamin
#3 curcuma
#4 omega
#5 phenols
#6 sugar or glucose
#7 calcium
#8 spice or saline or salt OR sodium
#9 #1 AND (#2 OR #3 OR #4 OR #5 OR #6 OR #7 or #8)

3) In Embase

#1 'osteoarthritis'/exp OR osteoarthritis OR 'osteoarthrosis'/exp OR osteoarthrosis
#2 vitamins OR vitamin
#3 curcuma
#4 omega3 or omega6
#5 phenols
#6 sugar or glucose
#7 calcium
#8 spice or saline or salt OR sodium
#9 #1 AND (#2 OR #3 OR #4 OR #5 OR #6 OR #7 or #8)

#9 AND ('arthralgia'/dm OR 'arthritis'/dm OR 'arthropathy'/dm OR 'cardiovascular disease'/dm OR 'cartilage degeneration'/dm OR 'chronic pain'/dm OR 'hip osteoarthritis'/dm OR 'knee osteoarthritis'/dm OR 'knee pain'/dm OR 'low back pain'/dm OR 'musculoskeletal disease'/dm OR 'osteoarthritis'/dm OR 'pain'/dm OR 'rheumatic disease'/dm OR 'spondylosis'/dm OR 'synovitis'/dm OR 'vitamin d deficiency'/dm) AND ('Article'/it OR 'Article in Press'/it OR 'Conference Abstract'/it OR 'Conference Paper'/it OR 'Letter'/it OR 'Short Survey'/it) AND ([adult]/lim OR [aged]/lim OR [middle aged]/lim OR [very elderly]/lim OR [young adult]/lim) AND ('case control study'/de OR 'case report'/de OR 'clinical article'/de OR 'clinical trial'/de OR 'cohort analysis'/de OR 'comparative effectiveness'/de OR 'comparative study'/de OR 'control group'/de OR 'controlled clinical trial'/de OR 'controlled study'/de OR 'cross sectional study'/de OR 'diagnostic test accuracy study'/de OR 'double blind procedure'/de OR 'human'/de OR 'longitudinal study'/de OR 'major clinical study'/de OR 'meta analysis'/de OR 'multicenter study'/de OR 'normal human'/de OR 'observational study'/de

OR 'open study'/de OR 'phase 2 clinical trial'/de OR 'phase 3 clinical trial'/de OR 'pilot study'/de OR 'practice guideline'/de OR 'prospective study'/de OR 'questionnaire'/de OR 'randomized controlled trial'/de OR 'randomized controlled trial topic'/de OR 'retrospective study'/de OR 'single blind procedure'/de OR 'systematic review'/de) AND ('25 hydroxyvitamin d'/dd OR 'c reactive protein'/dd OR 'calcium'/dd OR 'calcium phosphate'/dd OR 'colecalciferol'/dd OR 'endogenous compound'/dd OR 'glucose'/dd OR 'insulin'/dd OR 'placebo'/dd OR 'protein'/dd OR 'sodium'/dd OR 'sodium chloride'/dd OR 'triacylglycerol'/dd OR 'unclassified drug'/dd OR 'vitamin'/dd OR 'vitamin d'/dd)

Table S1. Population baseline characteristics of the studies included in the meta-analysis.

Study	Country	Intervention	Intervention group			Control group		
			Age (years)	Disease duration (years)	Pain intensity	Age (years)	Disease duration (years)	Pain intensity
**Atabaki [25]**	Iran	Curcumin	49.1	4.5	7.9	48.3	4.7	8.5
**Haroyan [26]**	Armenia	Curcumin	54.7	NA	5.9	56.0	NA	5.9
**Henrotin [27]**	Belgium	Curcumin	60.9	NA	NA	63.3	NA	NA
**Khanna [28]**	India	Curcumin	53.4	NA	7.0	51.5	NA	6.6
**Kuptniratsaikul 2014 [29]**	Thailand	Curcumin	60.3	NA	5.3	60.9	NA	5.4
**Kuptniratsaikul 2009 [30]**	Thailand	Curcumin	61.4	NA	5.3	60	NA	5.0
**Madhu [31]**	India	Curcumin	56.6	NA	6.6	56.8	NA	6.2
**Panda [32]**	India	Curcumin	55.2	NA	5.2	53.1	NA	5.3
**Shep [33]**	India	Curcumin	52.6	7.4	7.9	52.1	7.5	7.8
**Singhal [34]**	India	Curcumin	53.1	NA	NA	50.8	NA	NA
**Srivastava [35]**	India	Curcumin	50.2	NA	7.9	50.3	NA	7.7
**Wang [36]**	Tasmania	Curcumin	61.3	NA	5.6	62.4	NA	5.4
**Panahi [37]**	Iran	Curcumin	57.3	NA	6.6	57.6	NA	5.9
**Altman [38]**	USA	Ginger	64.0	7.0	5.0	66.3	7.0	5.0
**Wigler [39]**	Israel	Ginger	64.7	8.0	7.6	59.3	5.9	7.7
**Bolognesi [40]**	Italy	Ginger	52.3	NA	1.6	53.1	NA	1.5
**Jin [41]**	Australia	Vitamin D3	63.5	NA	4.9	62.9	NA	4.6
**MacAlindon [42]**	USA	Vitamin D3	61.8	NA	6.9	63.0	NA	5.8
**Sanghi [43]**	India	Vitamin D3	53.2	NA	7.5	53.0	NA	7.5
**Medhi [44]**	India	Vitamin C and E	54.8	NA	9.5	52.8	NA	9.2
**Dehghan [45]**	Iran	Vitamin E	47.5	NA	2.4	46.8	NA	2.5
**Tantavisut [46]**	Thailand	Vitamin E	69.5	NA	7.7	69.2	NA	8.8
**Wluka [47]**	Australia	Vitamin E	64.3	NA	4.7	63.7	NA	4.8
**Essouiri [48]**	Morocco	Vitamin E	58.2	4.5	NA	58.9	4.8	NA
**Colker [49]**	USA	Multivitamin	51.5	NA	4.4	59.0	NA	4.2
**Frestedt [50]**	USA	Multivitamin	58.5	NA	5.7	58.9	NA	5.0
**Jacquet [51]**	France	Omega-3	56.8	NA	NA	57.5	NA	NA
**Stammers [52]**	UK	Omega-3	67.0	14.0	NA	69.0	17.0	NA
**Chopra [53]**	India	Herbal	59.0	NA	6.17	55.0	NA	6.5
**Guo [54]**	China	Herbal	NA	NA	5.8	NA	NA	5.9
**Tao [55]**	China	Herbal	62.1	NA	6.6	64.0	NA	6.5

**Wu [56]**	China	Herbal	63.5	NA	5.7	63.0	NA	5.9
**Farpour [57]**	Iran	Herbal	47.1	NA	7.1	55.9	NA	7.8
**Gupta [58]**	India	Herbal	53.5	NA	5.8	51.9	NA	5.9
**Hamblin [59]**	UK	Herbal	NA	NA	4.5	NA	NA	2.4
**Karimifar [60]**	Iran	Herbal	52.0	NA	7.0	53.0	NA	7.0
**Karlapundi [61]**	India	Herbal	50.8	NA	5.8	50.3	NA	5.8
**Koonrungsesomboon [62]**	Thailand	Herbal	62.0	4.9	6.0	60.3	4.7	6.2
**Moré [63]**	Germany	Herbal	57.9	NA	6.0	55.7	NA	6.0
**Thomford [64]**	Ghana	Herbal	65.8	NA	NA	64.3	NA	NA
**Kakatum [65]**	Thailand	Herbal	58.5	NA	5.8	59.0	NA	5.5
**Pinsornsak [66]**	Thailand	Herbal	60.4	NA	4.4	58.2	NA	4.4
**Liu [67]**	Australia	Herbal	65.1	NA	5.9	66.2	NA	6.0
**Schumacher [68]**	USA	Cherry	NA	NA	NA	NA	NA	NA
**Puente [69]**	Cuba	Beeswax	67	NA	8.7	67.0	NA	9.2
**Shin [70]**	Korea	Deer bone	57.7	NA	3.8	59.9	NA	3.6
**Salimzadeh [71]**	Iran	Garlic	75.3	NA	8.3	76.6	NA	9.6
**Lau [72]**	China	Green mussel	62.1	10.7	6.0	62.9	7.1	6.0
**Zawadzki [73]**	Poland	Green mussel	65.6	NA	6.6	66.7	NA	6.6
**May [74]**	Malaysia	Bitter melon	62.0	NA	NA	57.8	NA	NA
**Sadat [75]**	Iran	Sesame seed	NA	4.1	9.5	NA	5.2	9.0
**Schell [76]**	USA	Strawberries	NA	NA	1.4	NA	NA	1.1

NA = not available

Figure S1. Forest plots of effects of vitamin D on OA parameters

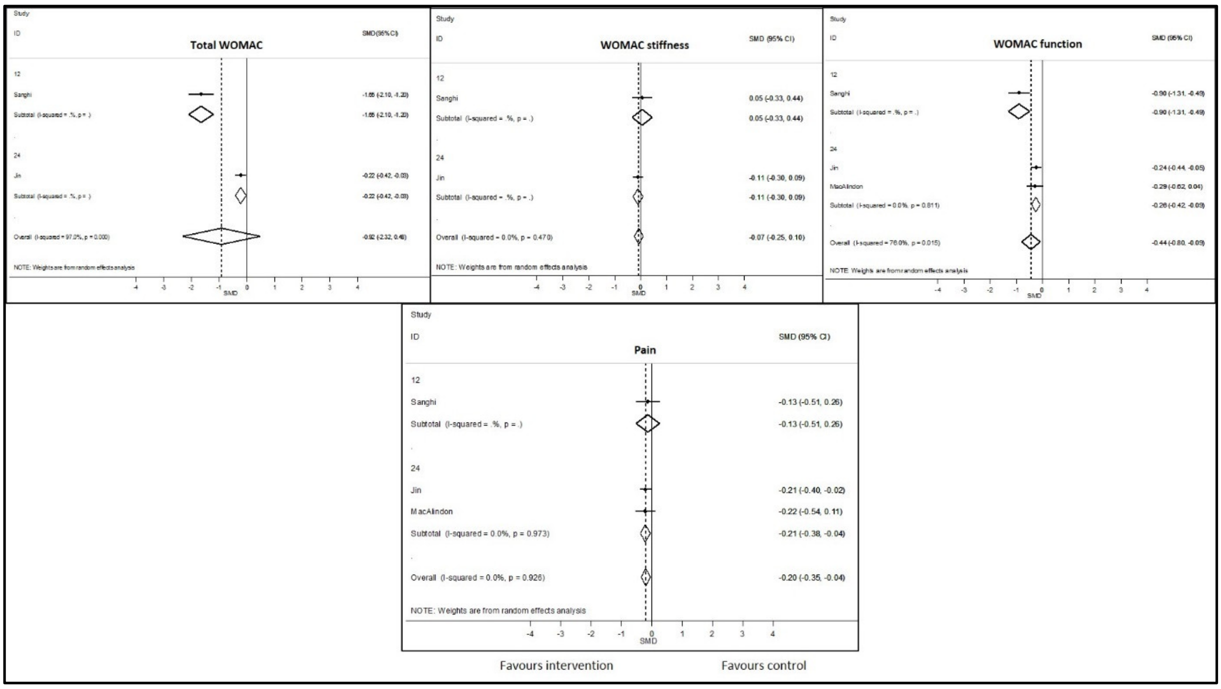

OA= osteoarthritis; WOMAC= Western Ontario and McMaster Universities Osteoarthritis Index; SMD= standardized mean difference; ID= study identity.

Figure S2. Forest plots of effects of curcumin on OA parameters other than pain

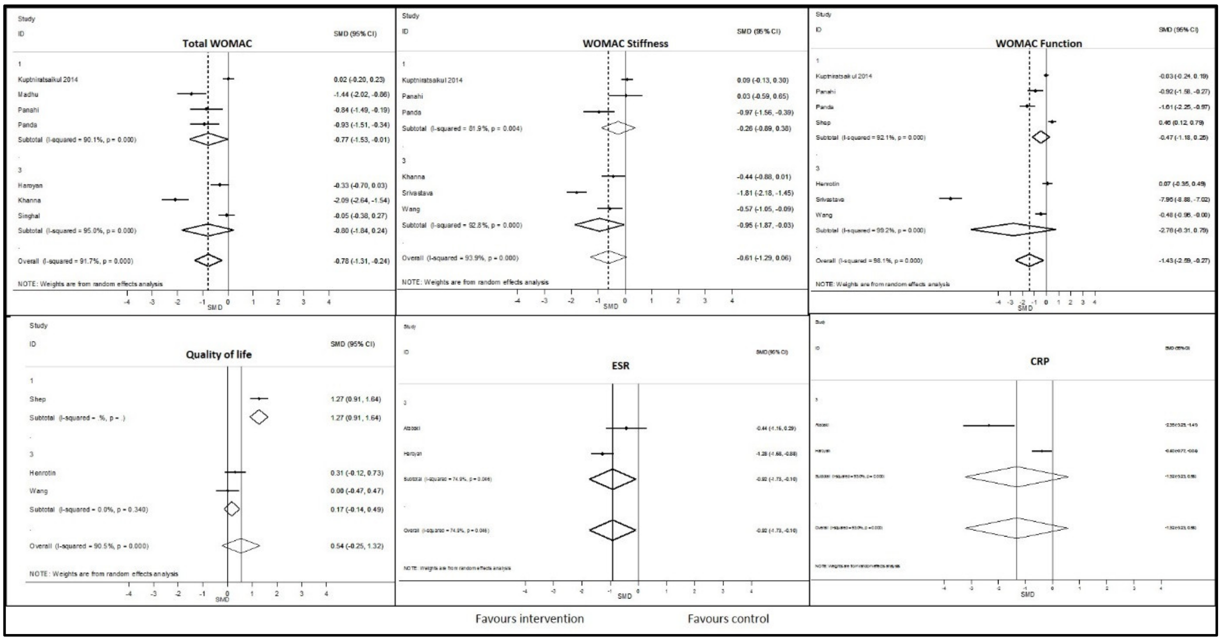

OA= osteoarthritis; WOMAC= Western Ontario and McMaster Universities Osteoarthritis Index; SMD= standardized mean difference; ID= study identity. ESR=erythrocyte sedimentation rate; CRP= C reactive protein

Figure S3. Funnel plots for sensitivity analysis

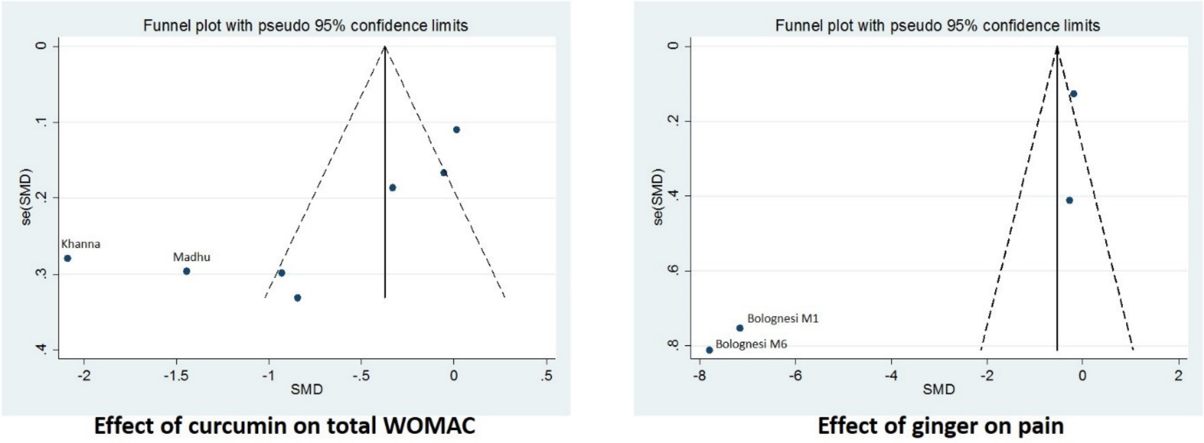

WOMAC= WOMAC= Western Ontario and McMaster Universities Osteoarthritis Index; SMD= standardized mean difference; M1= at 1 month; M6= at 6 months.

Figure S4. Forest plots of effects of ginger on OA parameters

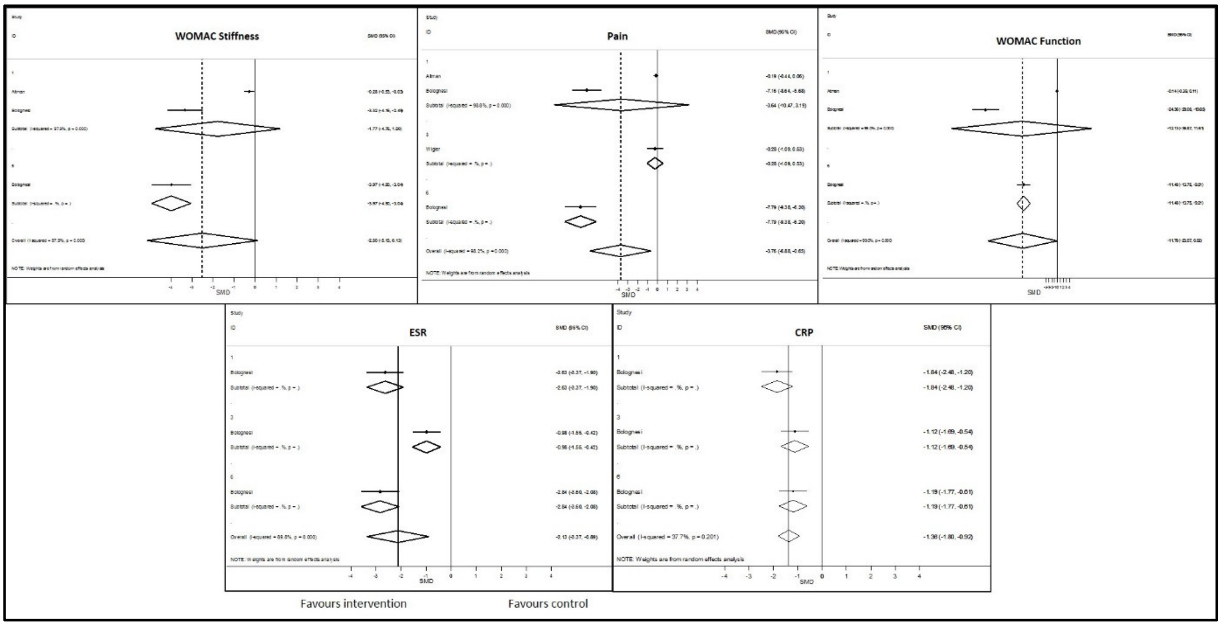

OA= osteoarthritis; WOMAC= Western Ontario and McMaster Universities Osteoarthritis Index; SMD= standardized mean difference; ID= study identity. ESR=erythrocyte sedimentation rate; CRP= C reactive protein

Figure S5. Forest plots of effects of vitamin E on OA parameters

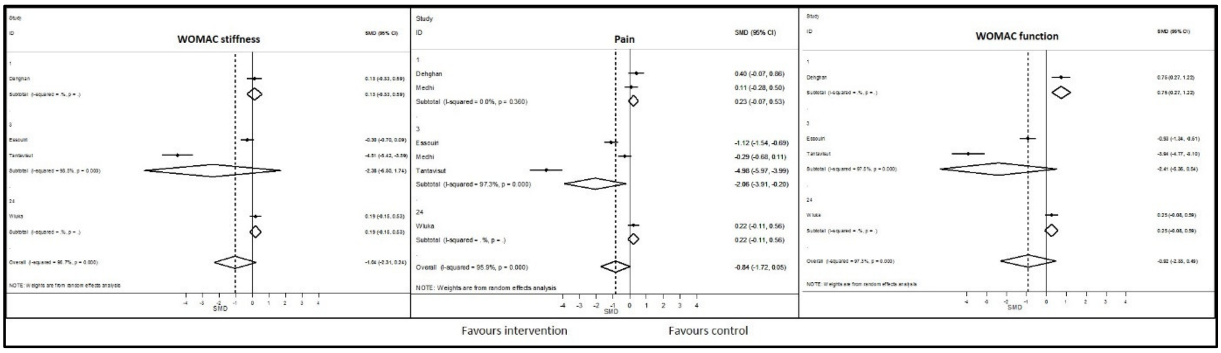

OA= osteoarthritis; WOMAC= Western Ontario and McMaster Universities Osteoarthritis Index; SMD= standardized mean difference; ID= study identity.

Figure S6. Forest plots of effects of herbal formulations on OA parameters

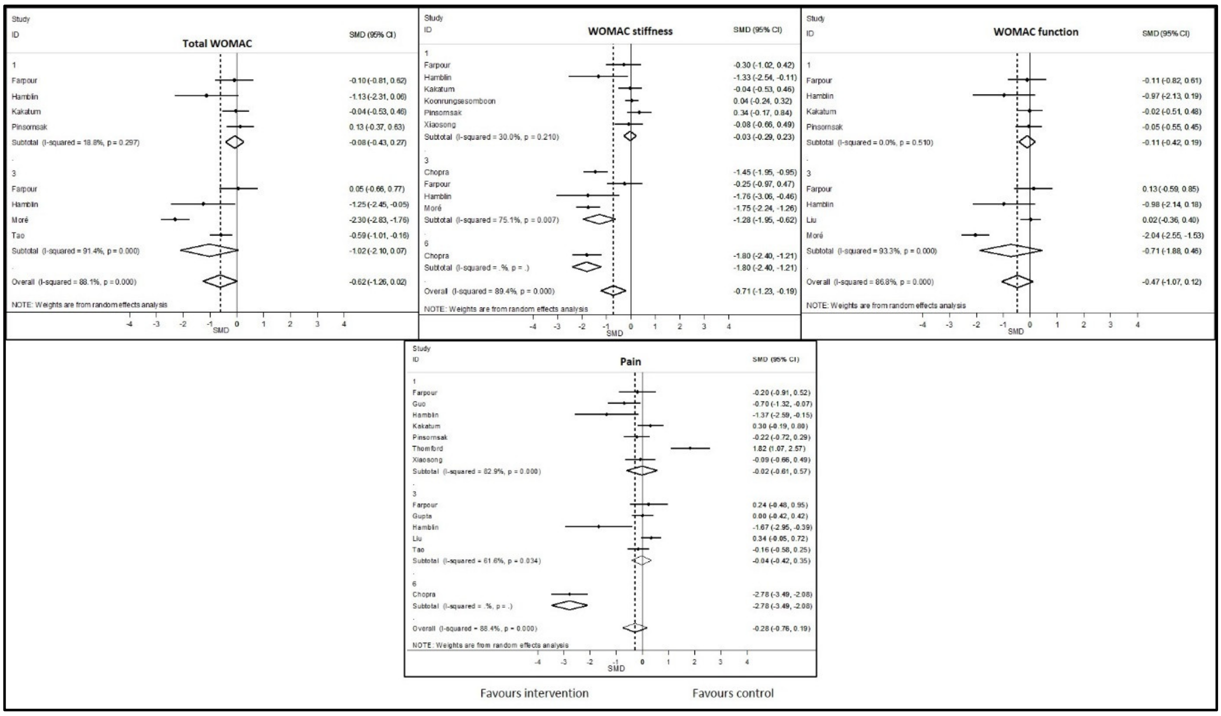

OA= osteoarthritis; WOMAC= Western Ontario and McMaster Universities Osteoarthritis Index; SMD= standardized mean difference; ID= study identity.
